# Global publications on end-tidal carbon dioxide: A bibliometric analysis

**DOI:** 10.1097/MD.0000000000046818

**Published:** 2026-01-02

**Authors:** Wenqin Wang, Duoqin Huang, Zixin Luo, Zhiyuan Zhang, Li Xiao, Xin Liu, Linlin Yue, Songmao Ouyang, Hongquan Zhu, Kang Zou

**Affiliations:** a Department of Anesthesiology, Affiliated Hospital of Jiangxi University of Traditional Chinese Medicine, Nanchang City, Jiangxi Province, The People’s Republic of China; b The First Clinical Medical College, Gannan Medical University, Ganzhou City, Jiangxi Province, The People’s Republic of China; c Department of Rehabilitation Medicine, The First Affiliated Hospital of Gannan Medical University, Ganzhou City, Jiangxi Province, The People’s Republic of China; d Department of Critical Care Medicine, The First Affiliated Hospital of Gannan Medical University, Ganzhou City, Jiangxi Province, The People’s Republic of China.

**Keywords:** bibliometric, EtCO_2_, visualization analysis, VOSviewer, Web of Science

## Abstract

**Background:**

Bibliometric and visual analysis of end-tidal carbon dioxide (EtCO_2_) literature was conducted to clarify the current research hotpots and development trends, and provide new ideas for basic research and clinical diagnosis and treatment.

**Methods:**

Without any time limit, Science Citation Index Expanded, Social Sciences Citation Index, and Conference Proceedings Citation Index – Science databases from the Web of Science core collection were used as data sources, and bibliometric and VOSviewer software were used to visualize and analyze the literature in terms of authors, journals, countries, institutions, and their collaborative networks, as well as the keyword networks.

**Results:**

The final inclusion of 4442 EtCO_2_-related publications, the number of publications is generally on the rise; the top 3 countries in terms of publications are the United States, the United Kingdom, and Canada; the top 3 institutions are the University of Toronto, the University of British Columbia, and Harvard University; the journal with the largest number of publications is the Journal of Applied Physiology. Currently, research in this field mainly focuses on the definition of end-expiratory CO_2_ partial pressure, monitoring methods, monitoring indexes, and application disciplines; the influencing factors related to the application of EtCO_2_, its application in various diseases, and its relationship with obstructive sleep apnea are the hot spots of research, and perhaps a major trend in the future.

**Conclusion:**

An increasing number of publications indicate that researchers are showing interest in the field of EtCO_2_, and ongoing research maintains a relatively mature level. The international community has established a good foundation for cooperation, and it is necessary to increase cooperation among researchers, institutions, and countries. At the same time, it is necessary to explore in-depth and strengthen personnel cooperation, expand the coverage of fund support, and further improve the quality of literature.

## 1. Introduction

End-tidal carbon dioxide (EtCO_2_) monitoring is a novel monitoring technique in current practice, which is a noninvasive method for measuring the concentration of carbon dioxide at the end of exhalation.^[1]^ EtCO_2_ is an important respiratory parameter that reflects not only ventilation function but also circulatory function and pulmonary blood flow. The principle of EtCO_2_ monitoring is based on the absorption of carbon dioxide at a wavelength of 4.26 μm by infrared light, and the concentration of carbon dioxide is calculated by monitoring the attenuation intensity of the infrared light, which is commonly used in absorbance spectrophotometry.^[2,3]^ In the critical care clinical setting, this noninvasive, early, real-time, simple, continuous, and rapid monitoring technique is beneficial for accurate assessment of the patient’s condition, leading to improved patient outcomes. The values provided by the EtCO_2_ monitor represent the end-tidal CO_2_ pressure. Therefore, end-tidal CO_2_ pressure is considered a noninvasive indicator of alveolar ventilation and is highly correlated with arterial CO_2_ partial pressure under physiological conditions.^[4]^ EtCO_2_ monitoring can continuously reflect respiratory function, and when patients experience abnormal conditions such as tachypnea, shallow and rapid breathing, or respiratory pauses, continuous dynamic monitoring of EtCO_2_ changes can detect these issues early. In the intensive care unit, it can be used to assess the effectiveness of mechanical ventilation by monitoring changes in EtCO_2_ concentration, which can determine whether the desired carbon dioxide elimination is achieved, guide the setting of ventilator parameters, ensure ventilation quality, and detect accidental extubation early, making it an essential tool for treating and monitoring critically ill patients^[5]^; in modern anesthesia monitoring, EtCO_2_ is not only a marker of successful intubation but also an important indicator of the patient’s respiratory and circulatory system status. It has been widely used in surgical anesthesia monitoring and has broad clinical application prospects.^[6]^

Bibliometric analysis is a comprehensive system and method that applies statistical methods to quantitatively analyze all data. It can effectively organize and analyze research topics, hotspots, and development trends in a systematic manner.^[7]^ Currently, with the rapid development in the field of EtCO_2_, traditional methods of literature review are no longer sufficient. Review articles often rely on subjective analysis. Therefore, this study aims to objectively, quantitatively, and systematically present the relevant research on EtCO_2_ through bibliometric analysis. By using VOS viewer software and bibliometric websites, publication trends, keywords, authors, institutions, collaborations, and co-citations can be visually analyzed. This analysis helps identify research trends and hotspots in the field, providing guidance for scholars in selecting research directions and offering new insights for basic research and clinical diagnosis and treatment.^[8]^

## 2. Materials and methods

### 2.1. Search strategy and data source

**Figure 1.**
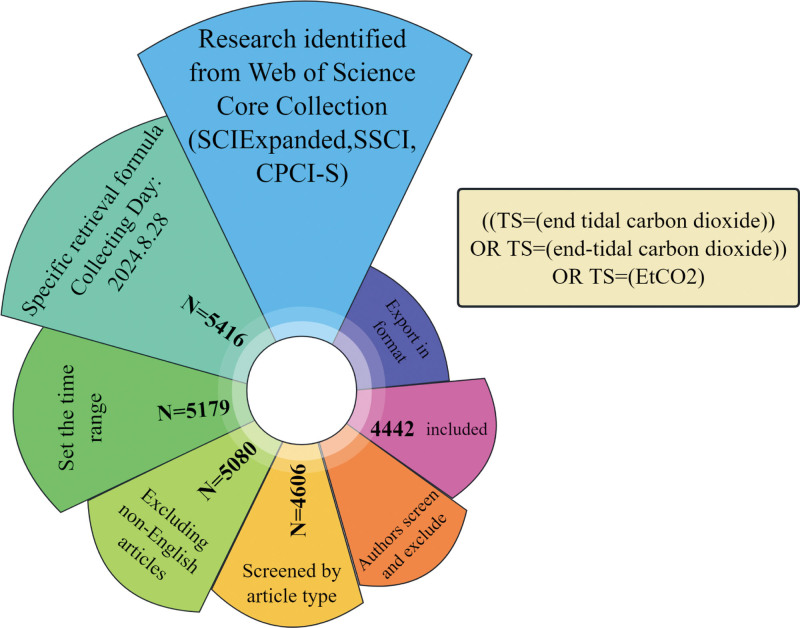
Rose flow chart of the screening process.

The Web of Science database is a comprehensive database covering multiple fields and is internationally recognized as an authoritative academic database in the natural sciences. The data source for this study is based on selecting the Science Citation Index Expanded, Social Sciences Citation Index, and Conference Proceedings Citation Index – Science citation indexes in the Web of Science core database. To more accurately find the target literature, we constructed an advanced search expression: ((TS = (end-tidal carbon dioxide)) OR TS = (end-tidal carbon dioxide)) OR TS = (EtCO_2_). To further determine the search scope, we limited the document types to “articles, reviews, and early access,” and the language to “English.” The time frame was chosen from the publication date of the 1st article to the same period in 2023. To ensure the quality of the literature, we limited the document type to academic journals and screened out duplicates and irrelevant literature. Two authors independently browsed all the literature and removed irrelevant literature. The 2 authors agreed on 99% of the literature, and the remaining literature was discussed with a 3rd author. We excluded 99 non-English literature articles, 474 non-compliant literature entries, and 164 duplicates or irrelevant studies. In total, 4442 articles were included in the analysis. The search date was August 28, 2024 (Fig. 1).

### 2.2. Data transformation and study methods

The selected literature was exported in 2 formats, “plain text file” and “tab-separated file,” and statistical analysis was conducted using software such as Bibliometrix, VOSviewer, and Citespace to summarize and identify research trends in terms of publication count, authors, countries, institutions, citation status, and keywords. Descriptive statistical analysis was performed using Excel to analyze the publication countries, institutions, and journals. Citespace 6.2.4 (Drexel University, Philadelphia) and VOSviewer 1.6.19 (Leiden University, Leiden, The Netherlands) were used to visualize and analyze collaboration networks, keywords, burst words, and other aspects of the literature. Bibliometrix was utilized for its aesthetic and detailed graphing capabilities to analyze authorship, countries, institutions, journals, and other networks. When conducting core analysis on authors, countries, and institutions, the Price theorem was employed for estimation. The Price theorem states that in a research field; the number of highly productive core authors is approximately equal to the square root of the total number of authors. Through further derivation, the formula for the minimum publication count of core authors in a field is determined as *M* ≈ 0.749 × (Nmax^1/2^), where Nmax represents the publication count of the most prolific scholar and *M* represents the minimum publication count for core authors in the field. Authors with a publication count greater than *M* are considered core authors in the field. Our analysis was conducted according to the BIBLIO literature metrology research guidelines.

## 3. Results

A total of 4442 articles were analyzed by 18,750 authors from 3637 institutions in 77 countries. These articles were published in 880 journals, with a total of 79,756 citations derived from 10,590 journals.

### 3.1. Analysis of the publications

**Figure 2.**
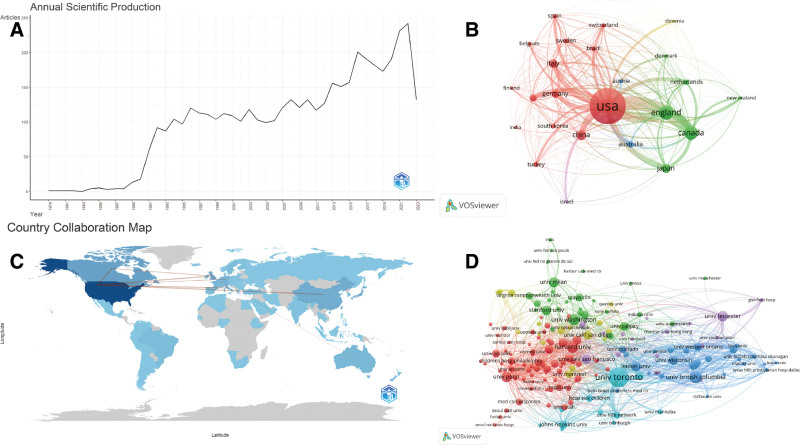
Annual publication volume and citations, as well as the analysis maps of international and institutional collaboration networks. (A) Annual publication volume and citations from 1979 to 2023. (B) Map of collaboration networks across core countries. (C) The visualization map of the published national cooperative geographic network. (D) Map of cooperation network of core issuing organizations.

As shown in Figure 2A, the total number of publications has steadily increased from 1979 to 2023, with a total of 4442 articles published and an annual growth rate of 11.24%. The highest number of publications in a single year was recorded in 2022 (=242, 5.45%). The 1st paper we found on Web of Science was published in 1979 by David Hurter et al.^[9]^ Overall, from 1998 to 2018, the number of publications showed a growing trend with occasional fluctuations in a few years. Additionally, this trend can be divided into 4 stages: slow production from 1979 to 1989, rapid growth from 1990 to 1996, relatively stable growth rate from 1997 to 2011, and overall acceleration in production from 2012 to 2023, indicating that the current research in this field is relatively mature but still requires exploration.

### 3.2. Analysis of the countries

All publications related to EtCO_2_ (n = 4442) were sourced from 77 countries. Among them, the United States had the highest total publication (TP) output (TP = 1541, 34.69%), followed by the United Kingdom (TP = 441, 9.93%) and Canada (TP = 416, 9.37%). In terms of citation counts, the United States ranked 1st with 53,147 citations, followed by Canada and the United Kingdom. Among the top 10 countries in terms of publication output, Canada, Switzerland, and the United States had the highest average citation counts per article. The complete data can be found in Table 1. Figure 2B displays a collaborative network map among core countries. The map shows a total of 24 countries determined based on the Price theorem, with different colors representing different clusters, and the thickness of the connecting lines indicating the strength of collaboration. The United States has the strongest and widest collaboration network, followed by the United Kingdom and Canada. The strongest collaboration network was found between the United States, the United Kingdom, and Canada. To further visualize the geographic collaboration network among countries, we plotted the graph shown in Figure 2C, where darker colors indicate a higher number of publications, and deeper connections reveal closer collaborations.

**Table 1:** Publications in the 10 most productive countries.

Ranked by TP	Country	TP	TC	TC/TP	Proportion/%
1	USA	1541	53,147	34.49	34.69%
2	England	441	13,122	29.76	9.93%
3	Canada	416	15,648	37.62	9.37%
4	China	287	2790	9.72	6.46%
5	Japan	250	4227	16.91	5.63%
6	Italy	228	7840	34.39	5.13%
7	Germany	202	6336	31.37	4.55%
8	France	180	5452	30.29	4.05%
9	Australia	166	3962	23.87	3.74%
10	Netherlands	129	4673	36.22	2.90%

TC = total citations, TP = total publications.

### 3.3. Analysis of the organizations

The statistical results show that a total of 3637 research institutions have participated in research on EtCO_2_. Among the top 10 institutions in terms of publication output, most of them are from the United States, United Kingdom, and Canada. The top 3 institutions are the University of Toronto, the University of British Columbia, and Harvard University, with publication outputs of 120, 59, and 53 articles, respectively, making them highly productive institutions in the field of EtCO_2_ (Table 2). Among the top 10 institutions, the University of Toronto has the highest citation count with 6245 citations. Harvard University ranks 1st in terms of average citations per article, followed closely by the University of Washington, with average citation counts of 105.04 and 104.06, respectively. Among the 3637 institutions, there are a total of 170 core institutions that have published more than 9 articles and are recognized as key contributors. The collaboration between these institutions is mainly centered around the University of Toronto, the University of British Columbia, and Harvard University, maintaining strong and close collaborative relationships (Fig. 2D).

**Table 2:** Publications in the 10 most productive organizations.

Ranked by TP	Organizations	TP	TC	TC/TP	Proportion/%
1	University of Toronto	120	6245	52.04	2.70%
2	The University of British Columbia	59	2187	37.07	1.33%
3	Harvard University	53	5567	105.04	1.19%
4	University of Washington	50	5203	104.06	1.13%
5	University of Leicester	46	1271	27.63	1.04%
6	University of Pennsylvania	45	1972	43.82	1.01%
7	University of Milan	43	2425	56.40	0.97%
8	Leiden University	41	1953	47.63	0.92%
9	Stanford University	40	1622	40.55	0.90%
10	University of Wisconsin	38	2134	56.16	0.86%

There is an overlap in the output of papers across research organizations due to co-publications.

TC = total citations, TP = total publications.

### 3.4. Analysis of the authors

**Figure 3.**
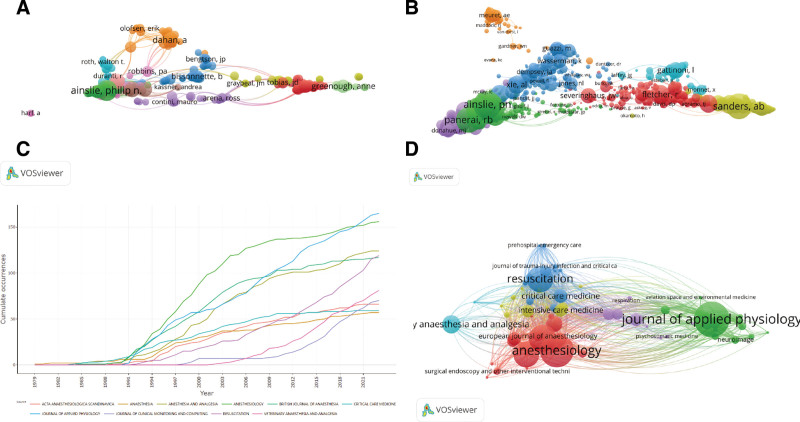
Visualization maps of authors, co-cited authors, and annual publication volume of journals. (A) Map of cooperation network of core issuing authors. (B) The visualization map of core co-cited authors. (C) Annual publication volume curve of the top 10 journals. (D) The visualization map of the core published journals.

The retrieved articles involve a total of 18,750 authors, with an average of 4.22 authors per article. The top 10 most prolific authors have contributed 223 articles, accounting for 5.02% of all published literature on EtCO_2_. Ainslie PN from the University of British Columbia has the highest number of publications with 44 articles, followed by Fisher JA from the University of Toronto and Panerai RB from Leicester University, both with 25 articles (Table 3). To explore the collaboration relationships among different authors, we used VOSviewer to visualize the author collaboration network, as shown in Figure 3A. For better visualization, the author collaboration network only includes 286 authors who have published at least 5 articles. Ainslie PN has the strongest collaboration with others. Co-cited authors refer to a group of authors who are simultaneously cited in 2 or more other papers, forming a co-citation relationship group that contributes to the subsequent development of the field. In the field of EtCO_2_ research, there are a total of 48,788 co-cited authors. The co-citation collaboration network among different authors in this field, limited to authors with more than 13 citations, is shown in Figure 3B.

**Table 3:** Publications in the 10 most productive authors.

Ranked by TP	Authors	TP	TC	TC/TP	Proportion/%	*H* index
1	Ainslie PN	44	1528	34.73	0.99%	65
2	Fisher JA	25	1158	46.32	0.56%	30
3	Panerai RB	25	361	14.44	0.56%	52
4	Dahan A	23	995	43.26	0.52%	73
5	Duffin J	21	694	33.05	0.47%	47
6	Greenough A	18	160	8.89	0.41%	58
7	Berkenbosch A	17	524	30.82	0.38%	25
8	Tymko MM	17	305	17.94	0.38%	23
9	Robinson TG	17	192	11.29	0.38%	63
10	Weil MH	16	1021	63.81	0.36%	73

There is an overlap in the output of papers across research authors due to co-publications.

TC = total citations, TP = total publications.

### 3.5. Analysis of the journals

From 1979 to 2023, a total of 4442 records were retrieved and published in 880 journals. Table 4 presents the top 10 journals in terms of publication output, accounting for 22.83% of the total publications. The journal with the highest output is the *《Journal of Applied Physiology》* with 165 relevant publications, representing 3.71% of the total records. The 2nd most significant journal is *《Anesthesiology》* with 156 publications. More than half of the top 10 prolific journals analyzed are in the field of anesthesia. According to the total citations, *《Anesthesiology》* has the highest total citations (8964), followed by *《Journal of Applied Physiology》* (6445) and *《New England Journal of Medicine》* (3838). Additionally, among the top 10 journals in terms of publication output, 6 of them belong to Q1, with *《Anesthesiology》* having the highest impact factor (10.7). To better understand the annual publication trends of relevant journals in the field, line graphs were created to analyze the annual publication growth of the top 10 journals, as shown in Figure 3C. Furthermore, based on Price theorem, 78 journals that published more than 10 articles were identified as core journals. The distribution of these core journals was visualized in Figure 3D to provide a more intuitive representation.

**Table 4:** Publications in the 10 most productive distribution.

Ranked by TP	Journals	TP	TC	TC/TP	IF2023/Q	Publishing place
1	Journal of Applied Physiology	165	6445	39.06	3.3/Q2	USA
2	Anesthesiology	156	8964	57.46	8.8/Q1	USA
3	Anesthesia and Analgesia	124	3802	30.66	5.7/Q1	USA
4	Resuscitation	119	2642	22.20	6.5/Q1	Ireland
5	British Journal of Anesthesia	117	3461	29.58	9.8/Q1	England
6	Veterinary Anesthesia and Analgesia	81	1036	12.79	1.7/Q2	England
7	Journal of Clinical Monitoring and Computing	70	660	9.43	2.2/Q4	Germany
8	Acta Anaesthesiologica Scandinavica	66	1150	17.42	2.1/Q4	Denmark
9	Critical Care Medicine	59	2396	40.61	8.8/Q1	USA
10	Anaesthesia	57	1861	32.65	10.7/Q1	England

IF2023/Q = Impact Factor 2023/Quartile, TC = total citations, TP = total publications.

### 3.6. Analysis of the keywords

**Figure 4.**
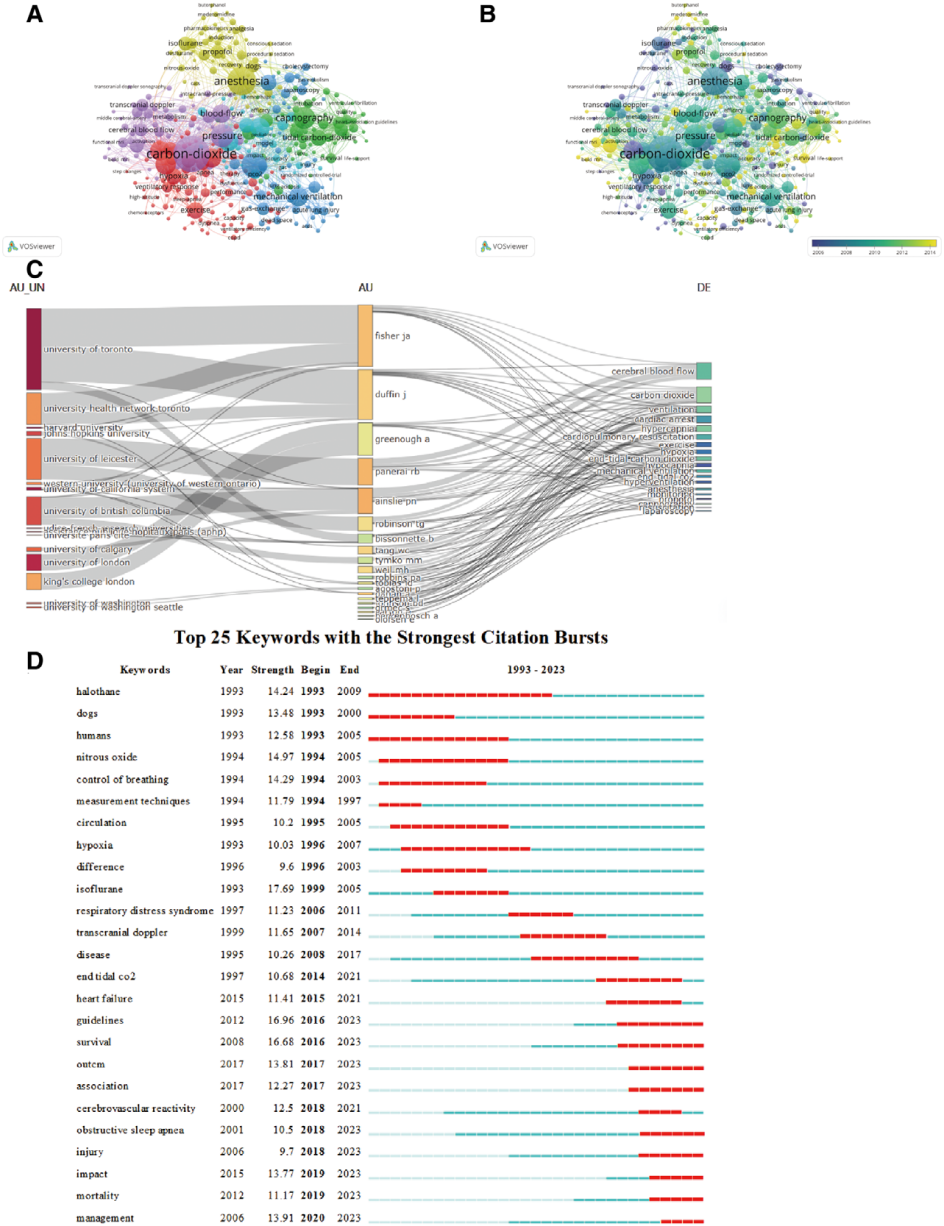
Visualization analysis of keywords and burst words. (A) Research on the visualization map of keywords clustering. (B) The visualization of the core keyword hotspots and trends. (C) Three-fields plot of authors, affiliations, and keywords in major EtCO_2_ papers. (D) Research on the burst period and intensity of burst words.

Keywords can often provide an intuitive representation of the themes and hot topics within a research field, with each selected keyword capturing the essence of an article. In the retrieved literature, there are a total of 12,011 keywords. Following Price law, we have constructed a network diagram of the main keywords with a frequency of occurrence >20, as shown in Figure 4A. The diagram consists of 346 nodes representing 346 keywords, with larger nodes indicating higher frequency of occurrence, and different colors representing different clusters. To gain a clearer understanding of the specific keywords, we have listed the top 20 keywords by frequency of occurrence in Table 5, which represent the main hot topics in EtCO_2_ research. From the clustering diagram, the keywords can be divided into 6 clusters. The results indicate that current research in this field primarily focuses on the definition of EtCO_2_, monitoring methods, monitoring indicators, and application disciplines. Cluster 1 (red) focuses on the relationship between diseases and behaviors and EtCO_2_, primarily investigating the value of EtCO_2_ in certain diseases, its application as a diagnostic, observational, and prognostic indicator for diseases, and the potential reactions and countermeasures that may occur after applying EtCO_2_ technology. Key keywords in this cluster include hypercapnia, obstructive sleep apnea, exercise, hypoxia, and oxygen. Cluster 2 (green) focuses on the application of EtCO_2_ in cardiopulmonary resuscitation, with keywords such as cardiopulmonary resuscitation, EtCO_2_, effectiveness, and survival rate. Cluster 3 (blue) explores EtCO_2_ monitoring in mechanically ventilated patients, with keywords including ventilation, mechanical ventilation, gas exchange, pressure, and lung injury. Cluster 4 (yellow) investigates the application of EtCO_2_ in the field of anesthesia, covering keywords such as anesthesia surgery, sedation, anesthesia, pain, propofol, inhalation, and analgesia. Cluster 5 (purple) focuses on the relationship between EtCO_2_ and hemodynamic changes, including keywords such as carbon dioxide, blood flow, pressure, blood flow rate, and blood flow velocity. Cluster 6 (cyan) delves into the application of EtCO_2_ in pediatrics, primarily targeting the pediatric population and discussing the differences compared to adult applications. To better illustrate the developmental process of this study, the clustering graph of keywords is overlaid with a timeline to generate a keyword timeline graph in Figure 4B. In addition, in order to clarify the main research content of the core authors, we screened some authors and drew the Three-Field Plot map of their institutions–author–keywords, as shown in Figure 4C.

**Table 5:** The top 10 high-frequency keywords.

Rank	Keywords	Occurrence	Rank	Keywords	Occurrence
1	Carbon dioxide	696	11	Mechanical ventilation	264
2	Anesthesia	496	12	Blood flow	262
3	Ventilation	411	13	Exercise	214
4	Pressure	359	14	Surgery	210
5	Capnography	338	15	Hypoxia	207
6	Hypercapnia	298	16	Tidal carbon dioxide	203
7	Humans	290	17	Arterial	184
8	Carbon dioxide	287	18	End-tidal CO_2_	180
9	Children	278	19	Cerebral-blood flow	175
10	CO_2_	273	20	Isoflurane	170

### 3.7. The keyword burst analysis

The keyword burst analysis refers to the analysis of the frequency and time distribution of keywords in the research field in a certain time node, and the selected frequency of sudden growth of keywords, and then can be used for the analysis of research hotspots, trends and frontier dynamic development and changes in this field. In order to have a clearer understanding of the research hotspots of the EtCO_2_ field, the Bursts function of Citespace can be further analyzed to obtain the influential nodes in each time section of the research field, and the emerging keywords ranked in the top 25 before the outbreak intensity can be selected, as shown in Figure 4D. The results showed that the keyword with the strongest outbreak intensity was “isoflurane” (the intensity was 17.69), and the relevant keywords, such as guidelines, survival rate and detection technology, were all reflected in the emergence of high intensity. In recent years, the continuous research hotspots are the influencing factors related to EtCO_2_ application, whether the technology is used in different diseases, and the relationship with obstructive sleep apnea, which indicates that recent research focuses on the possible negative effects of EtCO_2_ application and the analysis of related factors.

## 4. Discussion

### 4.1. Current research status

CO_2_, a byproduct of human metabolism, enters the lungs through the bloodstream and is exhaled. EtCO_2_ is an important respiratory parameter that reflects not only ventilation function but also circulatory function and pulmonary blood flow.^[10]^ Noninvasive EtCO_2_ monitoring has gained widespread attention in recent years and has been the subject of numerous clinical and experimental studies, both domestically and internationally, with significant achievements.^[11]^ The principle behind EtCO_2_ monitoring is that carbon dioxide is synthesized by tissue and organs, gradually transported to the lungs through capillaries and veins, and then eliminated from the body through respiration. Monitoring the EtCO_2_ partial pressure in exhaled breath can provide real-time and accurate reflection of arterial carbon dioxide partial pressure, allowing for a more intuitive understanding of pulmonary blood flow and ventilation conditions and the prevention of adverse events.^[12]^ Various portable EtCO_2_ monitoring devices have advantages such as rapid response, short warm-up time, real-time display of EtCO_2_ waveforms within 5 seconds, and comprehensive audio–visual alarm systems.^[13]^ In critical care clinical scenarios, this noninvasive, early, real-time, simple, continuous, and rapid monitoring technology is beneficial for accurate assessment of patient conditions, clinical decision-making, and ultimately improving patient outcomes.^[14]^ Bibliometric analysis allows for the evaluation of global academic output on EtCO_2_, aiding in decision-making and prioritization of limited resources.^[15]^ This study provides the 1st bibliometric analysis of global publications on EtCO_2_.

Analysis of the global publication quantity reveals an overall increasing trend in EtCO_2_-related research from 1979 to 2023, with occasional fluctuations, indicating sustained scholarly interest in EtCO_2_ research worldwide. Apart from the start and end dates of our study, 2 specific time points mark significant spikes in publications on EtCO_2_, namely 1990 and 2019. The occurrence of this phenomenon may be attributed to various factors. As Yang suggests, the overall growth in publications may be influenced by significant events occurring at specific time points.^[16]^ In 1988, Falk et al found that EtCO_2_ concentration decreased after cardiac arrest but increased and recovered during external chest compressions, indicating the restoration of spontaneous circulation.^[1]^ Subsequently, a large body of research focused on the application of EtCO_2_ monitoring in cardiopulmonary resuscitation, as well as the 1st proposal that EtCO_2_ is a noninvasive monitoring indicator of blood flow generated by chest compressions, which can be used to evaluate the effectiveness of chest compressions. Based on related research and findings, a peak in publications followed. In the 1990s, the scientific research field saw the emergence of many new technologies and methods, such as improvements in gas analysis instruments and advancements in computer simulations, making EtCO_2_ research more precise and feasible. In 2019, the global outbreak of the COVID-19 pandemic occurred. All these factors may have contributed to the increasing trend of global publications on EtCO_2_ to some extent. As of now, research in this field has reached a relatively mature stage, but there is still a need to explore innovative points and expand application scenarios.

A total of 77 countries has published research literature related to EtCO_2_, indicating global interest in this topic. In terms of publication quantity, the United States has been the largest contributor, accounting for 34.69% of the global publication output, which is more than 3 times that of the 2nd-ranked United Kingdom. Similar trends have been observed in other research directions in the fields of critical care and anesthesia, including artificial intelligence in intensive care,^[17]^ pulmonary arterial hypertension,^[18]^ and early mobilization in intensive care units.^[19]^ One potential reason for this phenomenon may be the abundant scientific funding and strong support for research, as well as the cultivation of scientific talents in the United States. In terms of average citations per article, the United States, United Kingdom, and Canada have higher numbers, while China has a lower average of 9.72. However, it is worth noting that China has made significant progress in scientific development in recent years, and the literature related to EtCO_2_ has also experienced explosive growth around 2015, indicating the need to focus not only on quantity but also on the quality of publications, actively explore innovative content, and publish more influential literature.

Articles related to EtCO_2_ have been published in 880 different journals, with the top 10 journals accounting for 22.83% of the total publications. Among the top 10 most productive journals, 6 belong to Journal Citation Reports Q1 (top quartile) and 2 belong to Q2 (2nd quartile), with 6 journals having an impact factor >5, indicating a high demand for research exploration in EtCO_2_ and high quality of most studies in this field. Furthermore, it was observed that most of the top journals are in the fields of critical care medicine and anesthesia. The distribution of these publications follows Bradford Law, indicating that most specialized topics are covered by a limited subset of specialized journals (such as critical care/anesthesia journals), some journals exist on the periphery of subject areas (e.g., nursing journals), and many others have broader, more general editorial scopes.^[20,21]^ The results of this study suggest that the core journals relevant to EtCO_2_ are those focused on critical care medicine and anesthesia, providing an important platform for publishing and exchanging research findings related to EtCO_2_.

From the perspective of publishing institutions, the top 10 institutions in terms of publication volume are all from Europe and America, with the University of Toronto having the highest publication volume. Different research institutions maintain high-intensity cooperative connections, presenting a multi-core and multi-cluster characteristic. However, although there are 170 core institutions, the publication volume of each institution is not large. The University of Toronto, which ranks 1st, has only 120 articles, and the University of British Columbia, which ranks 2nd, has less than half of the University of Toronto’s publications. Therefore, it is necessary to strengthen cooperation among universities and make contributions based on their respective strengths. In the core author group, many are international scholars, and the core group of collaborators led by Ainslie PN, Fisher JA, and Panerai RB has a large publication volume. Professor Ainslie PN has been focusing on the application of EtCO_2_ in fields such as stroke, cerebral autoregulation, and sports science, which is also a new research trend in recent years, with important guiding significance for the latest research. Professor Fisher JA, who follows closely, focuses on the application of EtCO_2_ in anesthesia and the correlation between EtCO_2_ and mechanical ventilation. Professor Panerai RB’s research also makes significant contributions in acute stroke, and his latest research shows that acute stroke patients are more prone to hypocapnia than the control group, supporting the value of routine CO_2_ assessment in acute stroke situations. Further research is needed to evaluate the clinical impact of these findings.^[22]^

Through cooperation, scholars who conduct research in the same field can not only share resources and exchange knowledge and ideas but are also crucial for the development of science and population health. Co-authorship analysis can clarify the frequency of different authors appearing in the same article to analyze the cooperative relationship between these authors. In EtCO_2_ research, we explored 18,750 authors, which means an average of 4.22 authors per article. However, we found that the number of core authors in this field is small, with only 286, and this ratio (286/18,750, 1.53%) presents a significantly different result from the national level (24/77, 31.17%). Moreover, most of these core authors come from Europe and America, and authors from other countries are rare. It is worth noting that we found that the entire core author cooperation network structure is relatively loose, with few relationships between authors from different countries, presenting a multi-center and multi-cluster type. However, this restricted cooperation network may exacerbate the polarization form, resulting in higher scientific output and influence in economically developed countries, while the scientific output and influence of relatively poor developing countries are lower.^[23]^ Therefore, we advocate strengthening cooperation with scholars from different countries, dedicating to the development of quality and diversity, and establishing cooperative relationships to obtain more innovative thinking as a solution to explore emerging and complex problems.

The interdisciplinary research paradigm provides a multidisciplinary perspective and approach to the study of EtCO_2_, and at the same time, we clearly emphasize that EtCO_2_ is a complex topic that needs to be explored by integrating the knowledge and methods of multiple disciplines. With the cross-fertilization of related monitoring technologies with various disciplines, its application value in the clinic will be more prominent. Due to the lack of clinical awareness of EtCO_2_ monitoring, the research potential of this technology is huge, and there is still a lot of work that deserves our in-depth exploration and research. In the follow-up study, we should further strengthen the cooperation and communication between different disciplines, form an interdisciplinary research team, and jointly explore the application of EtCO_2_ in multiple disciplines and fields.

### 4.2. Research hotspots and frontiers

Keywords clustering and time map shows that in the field of EtCO_2_ research mainly around its definition, monitoring, monitoring index, application discipline, the results confirmed that EtCO_2_ is a noninvasive monitoring index, its monitoring technology has been used in the clinical many diseases. The current application scope suggests that EtCO_2_ monitoring has high sensitivity, which is very suitable for the monitoring and use of surgical anesthesia, and has become a necessary routine monitoring means for clinical anesthesia monitoring. It can monitor ventilation,^[24]^ determine the position of endotracheal intubation,^[25,26]^ promptly detect mechanical failures of ventilators^[27]^ adjust parameters of ventilators,^[28,29]^ guide the removal of ventilators,^[30]^ and reflect circulatory function^[31]^ and pulmonary blood flow.^[32]^ Meanwhile, from the clustering, it is evident that EtCO_2_ is widely used for monitoring various diseases, such as chronic obstructive pulmonary disease,^[33]^ acute respiratory distress syndrome,^[34]^ pulmonary embolism,^[35]^ increased intracranial pressure,^[36,37]^ and obstructive sleep apnea.^[38]^ The temporal graph of keywords indicates that EtCO_2_ has gradually been applied in various disciplines, including but not limited to anesthesia,^[39]^ emergency medicine,^[40]^ intensive care,^[41]^ pediatrics,^[42]^ obstetrics, and gynecology.^[43]^

### 4.3. Application of EtCO_2_ in cardiopulmonary resuscitation (CPR)

Based on clustering results, Cluster II explores the application of EtCO_2_ in CPR emergencies, indicating that EtCO_2_ monitoring has become an essential tool for assessing and optimizing the quality of CPR. EtCO_2_ monitoring provides a noninvasive, real-time method to evaluate the patient’s ventilation status, circulation status, and alveolar ventilation/perfusion ratio, thereby guiding the implementation of CPR and assessing its effectiveness. During CPR, EtCO_2_ monitoring aids in determining the effectiveness of chest compressions and the recovery of spontaneous circulation. Studies have shown that EtCO_2_ can predict the alveolar–arterial difference in carbon dioxide by measuring the relationship between alveolar gas and arterial blood carbon dioxide content. The smaller the alveolar–arterial difference, the greater the systemic blood flow. The EtCO_2_ value is closely related to the quality of CPR, with higher EtCO_2_ values typically associated with a higher rate of return of spontaneous circulation. For instance, a study pointed out that a sudden increase in EtCO_2_ by more than 10 mm Hg may indicate the recovery of spontaneous circulation, thus avoiding unnecessary interruptions of chest compressions to repeatedly check the electrocardiogram during resuscitation. Additionally, EtCO_2_ monitoring can be used to assess the depth and frequency of chest compressions during CPR, as well as whether ventilation is appropriate.

The American Heart Association (AHA) recommends the use of EtCO_2_ monitoring as a method to assess the effectiveness of CPR (Class IIa recommendation), especially in adult basic/advanced life support.^[44]^ The 2020 AHA guidelines mention that EtCO_2_ monitoring should be used to improve the quality of CPR when conditions permit. This indicates that EtCO_2_ monitoring has become an indispensable part of CPR. The application of EtCO_2_ monitoring in CPR is also recommended in the “Expert Consensus on Emergency End-Tidal Carbon Dioxide Monitoring,” aiming to standardize and enhance the understanding and clinical application of EtCO_2_ monitoring in the field of emergency medicine. However, the reliability of EtCO_2_ as a prognostic marker still requires further research.^[45]^ Low EtCO_2_ values may be associated with poor prognosis, but this relationship is not always consistent. During CPR, EtCO_2_ monitoring should be considered in conjunction with other clinical information to make more accurate prognostic judgments. Moreover, the effectiveness of EtCO_2_ monitoring in more interventions or resuscitations still needs further research. Therefore, the application of EtCO_2_ monitoring in CPR holds broad prospects; it can not only improve the quality of CPR but also provide clinicians with important information about the patient’s condition.

Future research may further explore new applications of EtCO_2_ monitoring in CPR and enhance its accuracy in predicting patient outcomes. Firstly, future studies could focus on developing and refining novel real-time feedback systems and training models based on EtCO_2_ monitoring. These systems could provide immediate feedback during CPR, assisting rescue personnel in optimizing the depth and frequency of chest compressions. The AHA notes that the adjustment mechanism for CPR during in-hospital cardiac arrest can be based on thresholds of blood pressure and EtCO_2_. However, the feedback of these indicators is difficult to provide accurately to clinical physicians.^[46]^ The new CPR system, by monitoring EtCO_2_ levels in real-time, allows rescuers to adjust their compression techniques to ensure effective chest compressions, thereby improving the effectiveness of CPR and the survival rate of patients.

Secondly, EtCO_2_ monitoring may become an important tool for predicting the effectiveness of CPR and patient outcomes. High-quality CPR may help maintain the potential for patients to achieve good results and extend the time window for the implementation of advanced interventions. Studies have shown that, considering the duration and quality of CPR, the trajectory of EtCO_2_ at specific times may be an important predictive factor for outcomes in out-of-hospital cardiac arrest patients, for in-hospital prognostication.^[47]^ In the future, with the help of advanced information and communication technologies, EtCO_2_ readings at specific times could be instantly transmitted from the EtCO_2_ monitor to mobile devices. For each patient, predictive outcomes could be updated in real-time every minute and may not be limited to a specific point during the CPR process.^[48]^ However, it should be noted that the interpretation of EtCO_2_ in CPR is influenced by changes in ventilation and chest compressions during the process. Standardizing EtCO_2_ is more precise as it eliminates the impact of some factors that change during manual CPR, such as constant ventilation rate and compression depth.^[49]^ Based on this, its clinical application in guiding resuscitation deserves further research, which will help physicians make wiser clinical decisions during CPR and provide more personalized treatment plans for patients.

Thirdly, during CPR, the management of advanced airways is crucial for improving patient survival rates. EtCO_2_ monitoring can be used to confirm the position of endotracheal intubation and monitor the effectiveness of ventilation throughout the CPR process. It is recommended to use EtCO_2_ monitoring to determine the position of the intubation after endotracheal intubation. *The Chinese Expert Consensus on Emergency End-Tidal Carbon Dioxide Monitoring* points out that using a continuous monitoring EtCO_2_ monitor is the preferred method for determining the position of the tube after endotracheal intubation, superior to auscultation of the chest and X-ray imaging. Typically, observing a continuous stable waveform for more than 4 to 6 breaths can determine that the endotracheal tube is within the airway. However, note that this method cannot determine the depth of endotracheal intubation. Future research could focus on exploring new methods of EtCO_2_ monitoring in guiding advanced airway management, such as diagnosing airway obstruction or other ventilation issues by analyzing changes in EtCO_2_ waveforms, thereby improving the quality of airway management during CPR.

### 4.4. Application of EtCO_2_ in anesthesiology

Cluster Ⅳ focuses on the extensive application of EtCO_2_ in the field of anesthesiology, where its application value and research progress have always been a hot topic in anesthesiology research and clinical practice. Whether during the intraoperative period or postoperatively, EtCO_2_ monitoring plays a crucial role. In the early diagnosis and intervention during surgery, EtCO_2_ monitoring can reflect the patient’s ventilation status and circulatory function in real-time, which is vital for the timely diagnosis of severe allergic reactions, severe asthma, pulmonary artery perfusion disorders, and gas embolism.^[50]^ Rapid changes in EtCO_2_ can serve as a basis for early diagnosis. For instance, a rapid decrease in EtCO_2_ during severe allergic reactions can be a diagnostic marker, aiding in the timely implementation of treatment measures.^[51]^ EtCO_2_ is used to assess and monitor the patient’s ventilation, provide information about the state of pulmonary circulation and hemodynamics, detect and warn of complications such as impaired ventilation, pulmonary embolism, and pneumothorax, and offer opportunities for timely intervention. The application of EtCO_2_ monitoring in the postoperative recovery period also shows great potential. Intraoperative EtCO_2_ levels are associated with many postoperative adverse reactions or severe diseases, such as postoperative organ dysfunction,^[52]^ nausea and vomiting,^[53]^ and pulmonary complications.^[54]^ By continuously monitoring EtCO_2_, postoperative respiratory function abnormalities in patients can be detected in a timely manner, guiding the adjustment of respiratory support and reducing the occurrence of complications. EtCO_2_ monitors and bispectral index have been proven to be useful auxiliary means for monitoring the depth of sedation.^[55]^ By analyzing the trend and waveform characteristics of EtCO_2_ changes, combined with other physiological parameters, the depth of anesthesia can be more accurately judged, thereby optimizing the use of anesthetic drugs and reducing the discomfort and complications during the patient’s postoperative recovery period.

With the continuous advancement of science and technology and the development of clinical practice, EtCO_2_ monitoring technology has been upgraded and improved with the miniaturization of monitoring equipment, diversification of sampling methods, and precision of monitoring results. In the future, EtCO_2_ monitoring technology will become more popular and accurate, becoming one of the essential tools used by anesthesiologists. Therefore, the education and training of EtCO_2_ monitoring for anesthesiology medical staff should also be strengthened to ensure that medical personnel can correctly understand and apply EtCO_2_ monitoring results, thereby improving the quality of patient care. Based on this, the study by Phillip Atherton and others also indicates that capnography monitoring should be considered standard care for patients in the post-anesthesia care unit.^[56]^ The education and training of registered medical staff working in the post-anesthesia care unit are crucial before implementing EtCO_2_ or tcPCO_2_ monitoring. In summary, as a simple, noninvasive, and reliable monitoring indicator, EtCO_2_ provides valuable information for anesthesiologists, helping them ensure the safety of patients and the smooth progress of surgery.

### 4.5. Potential risks of EtCO_2_ monitoring

Research on the accuracy of EtCO_2_ monitoring data and the potential harm caused by its application has become a recent hot topic. Although we consider EtCO_2_ monitoring to be a noninvasive technique that provides a safe and reliable method for assessing ventilation and respiratory status by measuring carbon dioxide concentration in exhaled gases, like any medical procedure, it may also carry potential risks and complications. These risks are generally small and rare but may include:

Skin irritation: long-term use of adhesive sensors or masks for EtCO_2_ monitoring may cause skin irritation or allergic reactions in some individuals. In response, the development of new, convenient, and more comfortable devices has received widespread attention. Yedidia Blonder and colleagues, aiming to improve the tolerability of EtCO_2_ sampling lines used for capnography monitoring in non-intubated patients, have developed advanced sampling lines that reduce the device’s abnormal odors and incidents of skin redness or irritation, enhancing patient compliance with monitoring.^[57]^

Equipment-related complications: in rare cases, monitoring devices may experience technical issues such as sensor or tubing failure. Regular maintenance and calibration of the equipment help minimize the risk of such complications. Furthermore, accurate measurement of exhaled carbon dioxide levels is crucial for reliable EtCO_2_ monitoring. Equipment malfunction, improper calibration, or patient factors (such as airway obstruction) can lead to inaccurate readings. Close monitoring and troubleshooting help identify and resolve any discrepancies in the measurements, and healthcare professionals must remain vigilant in addressing any potential complications or inaccuracies that may arise during the EtCO_2_ monitoring process.

Risk of cross-infection: due to contact with the patient’s airway secretions, EtCO2 monitoring equipment, whether mainstream or sidestream carbon dioxide monitors, is at risk of contamination and cross-infection. Future strategies include the development of single-use sensors to reduce the risk of infection. Alternatively, for reusable devices and accessories, high-level cleaning and disinfection according to improved device sterilization methods should be conducted to prevent cross-infection.

As monitoring equipment becomes more compact, sampling methods more diverse, and monitoring results more precise, the application of EtCO_2_ monitoring technology will become even more widespread. Future technological developments may include more portable monitoring devices and more intuitive user interfaces to facilitate easier use in various clinical settings. At the same time, it is important to emphasize the importance and necessity of integrating multimodal monitoring. Combining EtCO_2_ monitoring with the continuous monitoring of other physiological parameters, including intracranial pressure, mean arterial blood pressure, cerebral perfusion pressure, heart rate, oxygen saturation, PtiO_2_, and tipH values,^[58]^ can achieve a more comprehensive patient assessment and management. This multimodal monitoring approach can provide richer information, assisting physicians in making more accurate clinical decisions. Furthermore, to ensure that healthcare professionals can correctly understand and apply EtCO_2_ monitoring results, future educational training will be enhanced. This may include online courses, simulation training, and hands-on practical training to improve the understanding and application capabilities of healthcare staff regarding EtCO_2_ monitoring. In conclusion, the benefits of conducting EtCO_2_ monitoring during anesthesia outweigh the potential risks, and it remains an important tool for ensuring patient safety and optimizing respiratory management during anesthesia.

The application of EtCO_2_ in obstructive sleep apnea is currently an emerging direction with fewer research articles, suggesting that future scholars can start from this entry point when conducting research, from which new mechanisms and breakthroughs can be explored. The latest research also points out that monitoring with EtCO_2_ has some value in determining obstructive sleep apnea syndrome condition. However, further clinical trials with multi-center and large samples are needed to explore and establish a unified judgment standard.^[59]^

Overall, the systematic analysis of the current global research landscape on EtCO_2_ presented in this study offers important implications for scientific policy-making, international collaboration, and future research directions. From a research policy perspective, the multidisciplinary application value of EtCO_2_ monitoring technology has been widely validated; however, its development remains uneven across regions. Developed countries, particularly the United States, dominate the field due to substantial research funding and strong talent support. Although developing countries have shown a significant increase in research output in recent years, the quality and influence of their work remain to be enhanced. Therefore, research policies should place greater emphasis on guiding and allocating resources toward high-quality and innovative studies, particularly in areas such as clinical translation, technological standardization, and primary-care implementation. Furthermore, the establishment of interdisciplinary collaboration platforms should be encouraged to integrate resources from anesthesiology, emergency medicine, critical care, nursing, and other related fields, so as to promote standardized application and widespread adoption of EtCO_2_ monitoring.

In terms of international collaboration, while multiple core research clusters have emerged in EtCO_2_ research, the collaborative network remains centered around developed countries in Europe and North America, with limited participation from developing nations. There is a critical need to strengthen cooperation in areas such as public health emergency response and the adaptation of medical technologies to resource-limited settings, thereby ensuring that EtCO_2_ monitoring can better contribute to global health equity.

Future research should focus on technological innovation, expansion of clinical applications, and multidimensional integration. On one hand, efforts should be directed toward developing more portable and intelligent EtCO_2_ monitoring devices, incorporating artificial intelligence algorithms for real-time data interpretation and early warning, thereby improving the convenience and accuracy of clinical operations. On the other hand, in-depth research on the application mechanisms of EtCO_2_ in emerging fields (such as obstructive sleep apnea, stroke management, and perioperative risk prediction) should be conducted. Additionally, multimodal monitoring integration (e.g., combining hemodynamic and cerebral oximetry parameters) should be explored to establish a more comprehensive patient assessment system. Moreover, enhanced training for healthcare professionals and public awareness education are essential to facilitate the translation of EtCO_2_ technological achievements into routine clinical practice, ultimately improving the overall quality and resilience of global healthcare services.

### 4.6. Limitations

In summary, this paper presents an analysis and visualization of the literature in the field of EtCO_2_ research, including overall publication characteristics, research content and hotspots, as well as trends, through the presentation of visual maps and tables. Compared to previous bibliometric studies, this research utilizes a greater number of visualization tools to present the research results from multiple perspectives in this field. However, there are certain limitations. Firstly, the data selection for citation analysis is based solely on English literature from the Science Citation Index Expanded, Social Sciences Citation Index, and Conference Proceedings Citation Index – Science core data in the Web of Science database, which may result in some important literature being overlooked from other databases or in other languages. However, the data on the source of funds is missing in the existing data set, which makes it difficult to analyze effective and scientific results. To some extent, this provides a scientific outlook for future research trends in this field, offering scholars exploring this field some references and guidance in terms of direction selection, team building, and method choices.

## 5. Conclusions

The bibliometric analysis of EtCO_2_ monitoring literature from 1979 to 2023 indicates a significant rise in scholarly interest globally. The United States, United Kingdom, and Canada lead in research output, with the University of Toronto, the University of British Columbia, and Harvard University being key contributors. The study’s focus on EtCO_2_’s definition, monitoring techniques, and clinical applications points to a dynamic field with potential for further interdisciplinary growth and international collaboration.

## Acknowledgments

The authors thank VOSviewer, CiteSpace, and bibliometric techniques for their support.

## Author contributions

**Conceptualization:** Wenqin Wang, Duoqin Huang, Kang Zou.

**Data curation:** Zixin Luo, Zhiyuan Zhang, Li Xiao, Xin Liu, Linlin Yue.

**Formal analysis:** Zixin Luo, Zhiyuan Zhang, Li Xiao, Xin Liu, Linlin Yue.

**Funding acquisition:** Kang Zou.

**Investigation:** Songmao Ouyang.

**Methodology:** Songmao Ouyang.

**Project administration:** Hongquan Zhu.

**Resources:** Hongquan Zhu.

**Software:** Hongquan Zhu.

**Supervision:** Hongquan Zhu.

**Validation:** Hongquan Zhu.

**Visualization:** Hongquan Zhu.

**Writing – original draft:** Wenqin Wang, Duoqin Huang, Kang Zou.

**Writing – review & editing:** Zixin Luo, Zhiyuan Zhang, Li Xiao, Xin Liu, Linlin Yue, Hongquan Zhu.
